# First-line dolutegravir/lamivudine penetrates lymph nodes and reduces HIV reservoirs comparably to triple therapy

**DOI:** 10.21203/rs.3.rs-9402443/v1

**Published:** 2026-06-03

**Authors:** Jose Molto, Elias Rosen, Stephen Bondoc, Lucía Bailón, Lidia Blay, Albert Caballero, Maria Garcia-Guerrero, Maria Puertas, Igor Moraes-Cardoso, Andrea Vazquez, Julià Blanco, Beatriz Mothe, Angela Kashuba, Jacob Estes, Javier Martinez-Picado

**Affiliations:** Fight Against Infections, Infectious Diseases Department, Hospital Germans Trias i Pujol; Eshelman School of Pharmacy, University of North Carolina at Chapel Hill, Chapel Hill, North Carolina, USA.; Vaccine and Gene Therapy Institute, Oregon Health & Science University, Beaverton, Oregon, USA.; Department of Infectious Diseases, Hospital Universitari Germans Trias i Pujol, Badalona, Spain.; Department of General Surgery, Hospital Universitari Germans Trias i Pujol, Badalona, Spain.; Department of General Surgery, Hospital Universitari Germans Trias i Pujol, Badalona, Spain.; IrsiCaixa; IrsiCaixa AIDS Research Institute; IrsiCaixa, Badalona, Barcelona, Spain.; Fundació Lluita contra les Infeccions, Badalona, Spain.; IrsiCaixa; IrsiCaixa, Hospital Universitari Germans Trias I Pujol; Eshelman School of Pharmacy, University of North Carolina at Chapel Hill, Chapel Hill, North Carolina, USA.; Oregon Health & Science University; IrsiCaixa

**Keywords:** HIV infection, antiretroviral drugs, drug distribution

## Abstract

Dual antiretroviral therapy with dolutegravir and lamivudine (DTG/3TC) is a recommended first-line regimen for people with HIV, but whether reduced-drug therapy maintains adequate antiviral pressure within lymph node (LN) reservoirs remains uncertain. In the DUALITY clinical trial, peripheral HIV-1 reservoir markers declined similarly in participants initiating DTG/3TC or dolutegravir-based triple therapy. Here we investigated viral persistence and antiretroviral drug distribution in LN using multimodal imaging. In 39 participants undergoing inguinal LN excision at baseline or during the first year of treatment, HIV-1 DNA and RNA were detected by DNAscope/RNAscope and antiretroviral spatial distribution was mapped by mass spectrometry imaging. HIV-1–infected cells declined rapidly after treatment initiation, with comparable reductions between treatment groups. Antiretrovirals were widely detected within LN tissue regions susceptible to containing HIV-positive cells. These findings provide spatial evidence that DTG/3TC achieves effective LN penetration and supports effective antiviral exposure within a key anatomical HIV reservoir.

## INTRODUCTION

Dual antiretroviral therapy (ART) with dolutegravir and lamivudine (DTG/3TC) has demonstrated durable virologic suppression, favorable safety, and long-term effectiveness comparable to three-drug regimens in both clinical trials and real-world settings.^1–3^ On the basis of this evidence, DTG/3TC is now recommended as a preferred first-line ART option for people with HIV (PWH).^4^ Despite its clinical success, questions remain regarding the biological adequacy of dual therapy with DTG/3TC in anatomical compartments that harbor persistent HIV reservoirs.

The DUALITY clinical trial compared HIV-1 reservoir dynamics in ART-naïve PWH initiating first-line ART with DTG/3TC (2DR) or a three-drug regimen consisting of dolutegravir plus emtricitabine/tenofovir alafenamide (DTG + FTC/TAF; 3DR). In addition to comparable plasma viral suppression, the study demonstrated similar reductions in peripheral blood markers of HIV-1 reservoir, including total and intact HIV-1 DNA, cell-associated HIV-1 RNA, and the inducible reservoir.^5^ However, peripheral blood measurements may not capture viral persistence within lymphoid tissues, particularly lymph nodes (LN), which are major contributors to the HIV reservoir.^6,7^

Lymph nodes present structural and biological barriers to antiretroviral drug delivery. Their complex architecture, fibrosis, and heterogeneous microenvironment can limit drug penetration and create gradients in antiretroviral exposure.^8–11^ These features raise the possibility that reduced drug pressure under dual therapy with DTG/3TC could permit ongoing viral persistence in this sanctuary site, even when systemic viral suppression is achieved.^12,13^ Traditional approaches used to quantify viral burden or drug concentrations in lymphoid tissue, such as bulk PCR or liquid chromatography–mass spectrometry (LC-MS/MS), provide limited spatial information into these processes and therefore cannot resolve the relationship between infected cells, antiretroviral exposure, and tissue structure.

Recent advances in imaging technologies enable spatially resolved analysis of viral persistence and drug distribution within complex tissues. In situ hybridization (ISH), immunohistochemistry (IHC), and mass spectrometry imaging (MSI) allow visualization of infected cells and antiretroviral drugs within intact lymphoid architecture.^9,11,14^ Here, we applied a multimodal imaging strategy to characterize HIV persistence and antiretroviral drug distribution in LN during the first year of ART initiation in participants enrolled in the DUALITY clinical trial. By integrating in situ detection of viral nucleic acids with spatial mapping of antiretroviral drugs, we investigated whether dual therapy with DTG/3TC achieves sufficient tissue penetration and local antiviral exposure within LN compared with triple therapy.

## RESULTS

### Study population and safety

Forty participants in the DUALITY trial initially consented to undergo a single ultrasound-guided inguinal LN excision during the study. Biopsy timepoints were randomly assigned within each study arm, at baseline (pre-ART) or at weeks 2, 4, 12 or 48 after ART initiation, with four participants representing each arm per timepoint. One participant from the 2DR group declined the procedure and was not replaced; therefore, 39 participants were included in the final analysis. Baseline characteristics are summarized in **Supplementary Table 1**. The study population consisted of young men, with a median (IQR) plasma viral load of 23,828 (4,921–50,045) copies/mL and CD4 + T cell count of 460 (364–568) cells/mm^3^ at study entry.

No severe complications were observed following LN excision. Mild seroma formation occurred in eight participants (20.5%), with the highest incidence observed early in the study (28.6%). This prompted a procedural review and implementation of a 48-hour post-biopsy compressive bandage, and following this modification, seroma incidence declined to 11.1%.

### HIV-1 DNA and RNA dynamics in lymph node

In situ hybridization analysis (ISH) of viral DNA (vDNA) and RNA (vRNA) was performed on subjacent LN sections using an adaptation of previously reported RNAscope protocols.^15,16^ Before ART initiation HIV-1 DNA-positive (vDNA+) and RNA-positive (vRNA+) cells were detectable in all evaluable LN samples (Table 1). At baseline, median (range) vDNA and vRNA levels were 2.29 (0.80–4.14) and 6.01 (0.62–38.41) positive cells/10^5^ cells in the 2DR group, and 8.51 (0.51–36.86) and 15.72 (0.62–38.41) positive cells/10^5^ cells in the 3DR group.

**Figure 1:**
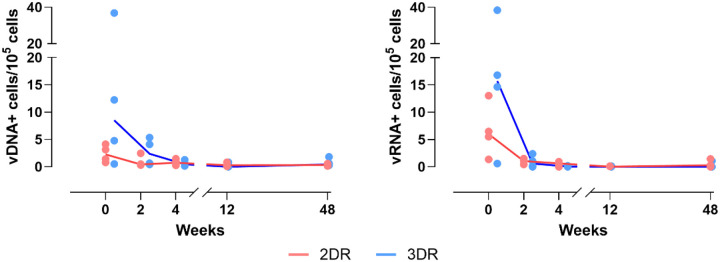
HIV-1 DNA- and RNA-positive cells in lymph nodes following antiretroviral treatment initiation. Lines represent medians and dots represent individual data.

Following ART initiation both vDNA + and vRNA+ cells in LN declined rapidly (Fig. 1). At week 48, the median fold change from baseline (95%CI) in vDNA+ cells was 0.158 (0.036–0.416) in the 2DR group and 0.055 (0.007–0.692) in the 3DR group. Corresponding fold changes in vRNA+ cells were 0.049 (0.002–0.532) and 0.001 (< 0.001–0.009), respectively. By week 48, vDNA + and vRNA+ cells in LN were of similar magnitude in both treatment arms (Table 1).

**Figure 2:**
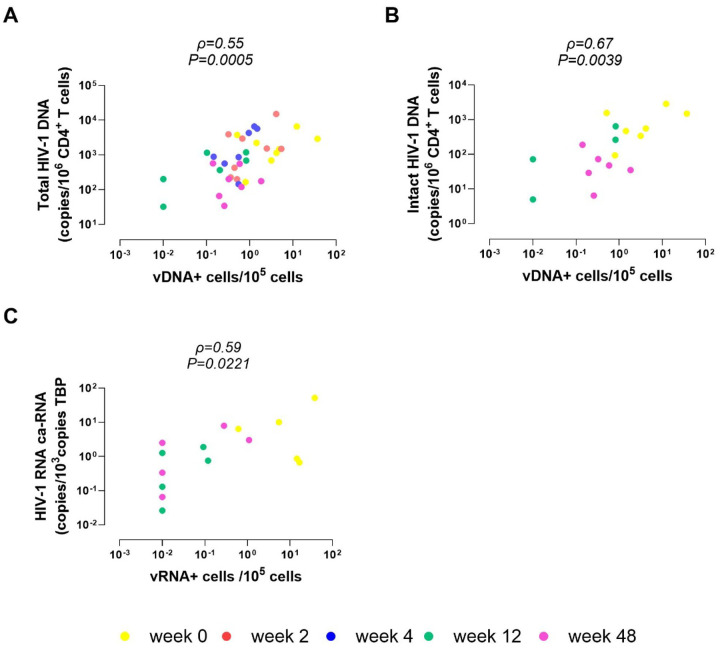
Correlation between HIV-1 reservoir measurements in peripheral blood CD4+ T cells and in lymph nodes. (A)Total HIV-1 DNA, (B) Intact HIV-1 DNA, (C) cell associated HIV-1 RNA. Spearman’s ρ and p-values are shown.

Levels of vDNA+ cells in LN correlated with total and intact HIV-1 DNA in peripheral blood CD4 + T cells measured at the same timepoints (r = 0.55, p = 0.0005; r = 0.67, p = 0.0039, respectively; Fig. 2). Similarly, the level of LN vRNA+ cells also correlated with peripheral blood cell-associated HIV-1 RNA (r = 0.59, p = 0.0221).

### Quantitative analysis of antiretrovirals concentrations in plasma and lymph node

Venous blood sampling was performed in parallel to LN excision, and concentrations of antiretrovirals (tenofovir [TFV], FTC, 3TC, DTG) in plasma and in LN tissue homogenates along with the intracellular metabolites tenofovir diphosphate (TFVdp), emtricitabine triphosphate (FTCtp), and lamivudine triphosphate (3TCtp) were quantified using validated LC-MS/MS methods.^14,17,18^ Paired LN biopsies and plasma samples were collected approximately 24 hours after dosing in 37 participants (median 26.7 hours, min-max 19.5–32.0 hours). Two participants sampled earlier post-dose were excluded from pharmacokinetic analyses, since drug concentrations were not representative of trough concentrations.

Antiretroviral concentrations in plasma and LN measured by LC-MS/MS, including parent drugs and phosphorylated active metabolites for nucleoside reverse transcriptase inhibitors (NRTI), are shown in Table 2. Plasma concentrations were consistent with previous reported 24-hour trough measurements.^8,19–22^ Median (range) LN-to-plasma tissue penetration ratios (TPR) indicated preferential tissue accumulation of TFV (4.34 [2.06–11.62]), FTC (1.58 [0.24–5.20]), and 3TC (1.46 [0.02–6.46]), but not DTG (0.18 [0.05–0.37]). Despite lower penetration ratio, DTG exhibited the highest absolute LN tissue concentrations (Table 2). Plasma and LN concentrations were strongly correlated for DTG (r = 0.819, p < 0.0001), with weaker correlations for NRTI (Table 2, **Supplementary Fig. 1**). Notably, the tissue metabolite-to-parent ratio in LN was higher for 3TC (4.19 [0.58–9.16]) than for FTC (1.59 [0.57–5.73]) or TFV (1.58 [0.57–4.94]), suggesting more efficient phosphorylation to the active metabolite of 3TC in LN tissue (Table 2).

### Spatial distribution of antiretrovirals in lymph node and colocalization with HIV-1 DNA- and RNA-positive cells

Spatial distribution of antiretrovirals within LN was assessed by Infrared Matrix-Assisted Laser Desorption Electrospray Ionization (IR-MALDESI) Mass Spectrometry Imaging (MSI).^9,11,23^ In MSI datasets, each measurement corresponds to a voxel (volumetric pixel), representing the three-dimensional tissue volume sampled by a single laser ablation event. Although LC-MS/MS confirmed the presence of TFV in LN samples, TFV concentrations measured by MSI were below the lower limit of quantitation (LLOQ) and were therefore not detectable by this method. As a result, spatial analyses of antiretroviral distribution and colocalization with vDNA + or vRNA+ cells focused on participants allocated to the 2DR group. Notably, because MSI LLOQ exceeded relevant in vitro IC90 values for both DTG and 3TC,^19,24–28^ locations where DTG or 3TC were measured had efficacious drug concentrations.

**Figure 3:**
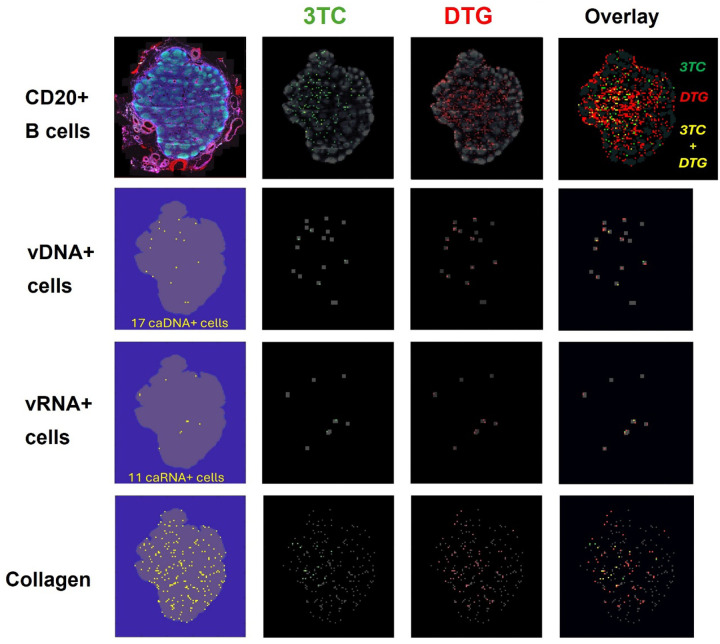
Representative spatial colocalization of HIV-1 vDNA- and vRNA-positive cells with antiretroviral drugs in lymph nodes (ID 295007, week 2)

**Figure 4:**
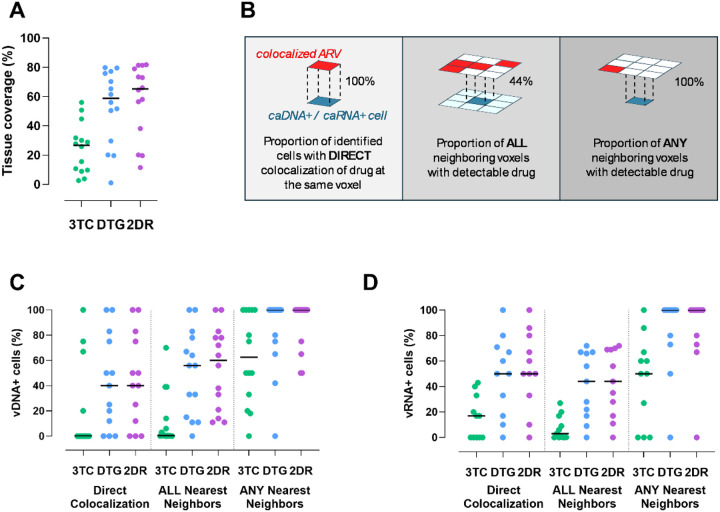
Lymph node antiretroviral coverage (A); approaches used to assess spatial distribution of antiretroviral drugs and HIV-positive cells (B); colocalization of antiretroviral drugs with HIV-1 DNA- (C) and vRNA-positive (D) cells in participants allocated to the 2DR group. Lines represent medians and dots represent individual data

DTG and 3TC were consistently detectable in LN tissue by MSI. Figure 3 displays representative spatial maps of DTG and 3TC for ID 295007 (2DR), collected at the week 2 timepoint. Total antiretroviral distributions are shown relative to CD20 expression of the LN B-cell follicles, from which it is evident that primary drug accumulation occurs within the extra-follicular T-cell zone or medulla. Drug distribution within LN tissue was heterogeneous, with median (range) relative standard deviations of 60% (36–77) for DTG and 43% (30–80) for 3TC. The proportion (median [range]) of LN tissue covered by at least one detectable antiretroviral was 65.3% (11.5–81.8), driven primarily by DTG (58.8% [1.1–79.9]). Notably, 32.0% (3.2–64.0) of LN tissue area was covered by combined DTG and 3TC, indicating that DTG/3TC was penetrating to a high extent within the LN (Fig. 4A).

DTG and 3TC distributions were coregistered with vDNA + and vRNA+ cells to assess spatial relationship between each target. This was done according to three different approaches (Fig. 4B): i) as the proportion of vDNA + or vRNA+ cells with direct colocalization of drugs at the same voxel; ii) as the proportion of all neighboring voxels with detectable drug; and, iii) as the proportion of vDNA + or vRNA+ cells with detectable drugs at any neighboring voxels. Representative colocalization between DTG and 3TC and vDNA + and vRNA+ cells for ID 295007 at week 2 can be seen in Fig. 3, where antiretroviral distributions have been isolated in regions containing vDNA + or vRNA+ cells and their nearest neighboring voxels to assess proximity of drug exposure. Colocalization results for DTG/3TC are summarized in Fig. 4C and D. Direct colocalization of DTG or 3TC with HIV-1 infected cells occurred in a median (range) of 45% (0–100) of vDNA+ cells and 50% (0–100) of vRNA+ cells, and, as with total tissue coverage, this was largely driven by DTG. When considering the proportion of sampling locations adjacent to infected cells with detectable antiretroviral drug, colocalization percentages were 60% (11–100) for vDNA+ cells and 44% (0–72) for vRNA+ cells. Notably, 100% (50–100) of vDNA+ cells and 100% (0–100) of vRNA+ cells had at least one neighboring voxel containing DTG or 3TC.

### Collagen deposition and antiretroviral drugs penetration in lymph node

Collagen deposition was measured in LN by IHC, as a marker of LN fibrosis. Overall, collagen I occupied a median (range) of 9% (2–19) of LN tissue area and did not show any consistent temporal changes following ART initiation. Collagen deposition was not associated with clinical parameters or peripheral blood reservoir measures (**Supplementary Fig. 2**).

Representative colocalization of collagen I and DTG or 3TC can be seen in Fig. 3. Quantitative imaging analysis from individuals allocated to the 2DR group found that colocalization between antiretrovirals and collagen (median [range] of 49% [9–77]) was lower than the total antiretroviral tissue coverage and that the majority of collagen colocalization occurred with DTG (45% [1–76]). Notably, greater collagen deposition in the LN was associated with lower DTG LN-to-plasma ratios, reduced tissue concentrations of DTG, and diminished antiretroviral coverage of LN tissue and HIV-infected cells (**Supplementary Fig. 3**), although these relationships did not reach statistical significance.

## DISCUSSION

In this sub-study of the DUALITY clinical trial, we provide an integrated spatial assessment of HIV-1 persistence and antiretroviral drug penetration in human LN during initiation of first-line ART with DTG/3TC. Using a multimodal approach combining ISH, MSI, and tissue pharmacokinetics, we show that HIV-infected cells within LN decline rapidly after ART initiation and that the magnitude and kinetics of reservoir decay are comparable between dual therapy with DTG/3TC and DTG-based 3DR. These findings extend our prior observations in peripheral blood and provide direct tissue-level evidence addressing concerns that dual ART with DTG/3TC may exert insufficient antiviral pressure within anatomical reservoir sites.

The rapid decline in vRNA + and vDNA+ cells observed in lymph nodes mirrored the early reductions detected in peripheral CD4 + T cells,^5,29^ supporting the notion that DTG-based regimens maintain potent antiviral activity across compartments enriched for long-lived infected cells. As described in prior HIV and SIV studies,^30–33^ vRNA declined more rapidly than vDNA, consistent with preferential clearance of transcriptionally active infected cells. By week 48, reservoir levels in lymph nodes were of similar magnitude in both treatment arms, and fold-change estimates suggested comparable reductions under dual and triple therapy, although the limited sample size precluded formal statistical comparisons. Together, these findings do not support the hypothetical caveat that reduced drug pressure with DTG/3TC would permit greater viral persistence in lymphoid tissues and instead reinforce evidence that dual therapy with DTG/3TC achieves reservoir decay comparable to triple therapy in ART-naïve individuals.

Our findings also have implications for the use of lymph node biopsies to study HIV reservoir dynamics. Lymph node sampling is an invasive procedure that requires surgical expertise and may be associated with complications such as seroma formation, bleeding, or infection. In this study, measures of HIV-1 persistence in lymph nodes correlated with corresponding determinations in peripheral blood CD4 + T cells, including total and intact HIV-1 DNA and cell-associated HIV-1 RNA. These observations suggest that peripheral blood measurements may partly reflect reservoir dynamics occurring within lymphoid tissues. Although lymph nodes remain a key anatomical site of HIV persistence and provide spatial insights that cannot be captured in blood, our results indicate that blood-based reservoir measurements may provide a useful surrogate for assessing global reservoir dynamics in clinical studies.

The biological adequacy of DTG/3TC within LN is further supported by our pharmacokinetic analyses. LC-MS/MS confirmed that all drugs reached the LN at concentrations near or exceeding relevant inhibitory thresholds,^19,34,35^ with DTG achieving the highest absolute concentrations despite lower LN-to-plasma ratios, likely reflecting its high degree of plasma protein binding. Consistent with previous work,^9,11^ MSI revealed substantial spatial heterogeneity in drug distribution across LN tissue, indicating the presence of local gradients in drug exposure. Nevertheless, DTG was present in approximately 60% of LN tissue, with combined DTG and 3TC coverage in one-third of the tissue area. Consistent with its higher lipophilicity, DTG was the primary driver of tissue and target-cell exposure.

Importantly, despite heterogeneous drug distribution, nearly all infected cells were located within regions containing detectable antiretroviral signal consistent with inhibitory concentrations in their immediate microenvironment. Although direct colocalization occurred in only 40–60% of infected cells, almost all vDNA + and vRNA+ cells had at least one neighboring voxel containing DTG or 3TC. Given that CD4 + T cells are highly motile in LNs, infected cells are likely migrating in and out of regions of high ARV exposure. These spatial findings indicate that DTG/3TC maintains sufficient local drug pressure even within the structural constraints of the LN. Compared with prior MSI studies in non-human primates using 4-drug ART regimens,^9,11^ our results show that DTG/3TC achieves equal or greater proximity to infected cells, likely reflecting favorable physicochemical properties and preferential distribution within extra-follicular T-cell zones, where most HIV-infected cells reside.

We also evaluated collagen I expression as a marker of LN fibrosis.^36,37^ Overall, collagen deposition was modest, stable over time, and comparable across participants. While no correlation was observed between collagen content and NRTI tissue concentrations, greater collagen deposition tended to correlate with lower DTG penetration and diminished ARV coverage of infected cells. Although these relationships did not reach statistical significance, likely reflecting limited sample size, their directionality is consistent with the hypothesis that fibrosis may restrict diffusion of certain antiretroviral drugs within lymphoid tissue.

This study is strengthened by its integrated, multimodal imaging approach, which enables simultaneous assessment of viral persistence, drug exposure, and tissue architecture within human LN during ART initiation. The collection of LN samples at multiple early timepoints offers unique insight into the kinetics of reservoir decay at the tissue level. These data are vital given the prevalence of infected cells within the LN and its potential role as a conduit for rapid viral rebound following treatment interruption. Therefore, these findings have implications not only for ART durability but also for strategies targeting tissue reservoirs in HIV remission and cure research.

Several limitations should also be considered. First, each participant contributed a single LN biopsy, preventing longitudinal within-subject analysis and limiting the ability to account for inter-individual variability. Second, the relatively small sample size restricted the power to evaluate correlations. Although the number of participants per time point was limited, this sampling design reflects the ethical and logistical constraints of LN excision studies in humans. Despite modest sample size, the consistency across virologic, pharmacokinetic, and spatial analyses supports the robustness of our findings. Finally, MSI lacked sensitivity for TFV, limiting spatial analyses in the 3DR arm and precluding full spatial assessment in those participants. The volume of material probed by IR-MALDESI at each sampling location across a tissue section is inherently much smaller than an LC-MS/MS tissue homogenate to preserve discrete spatial information, and there is a commensurate difference in analyte sensitivity.

In conclusion, this study provides compelling tissue-level evidence that dual ART with DTG/3TC achieves rapid suppression of HIV-infected cells within LN, with reservoir decay comparable to DTG + FTC/TAF. Despite heterogeneous tissue penetration, nearly all infected cells were exposed to inhibitory concentrations within their local microenvironment. These findings support the mechanistic plausibility of DTG/3TC as an effective first-line ART regimen and underscore its capacity to suppress HIV reservoirs within a key anatomical sanctuary.

## ONLINE METHODS

### Study Design and Objectives

DUALITY was a randomized, open-label clinical trial conducted in 44 ART–naïve PWH at the Infectious Diseases Department of Hospital Universitari Germans Trias i Pujol (Badalona, Spain). Participants were randomized (1:1) to initiate first-line ART with once-daily dolutegravir (DTG) 50 mg plus lamivudine (3TC) 300 mg (2DR group) or DTG 50 mg plus emtricitabine/tenofovir alafenamide 200/25 mg (DTG + FTC/TAF; 3DR group) and were followed for 48 weeks. Detailed trial design and primary outcomes have been reported previously.^5^

This sub-study aimed to characterize HIV-1 reservoir dynamics within peripheral LN during the first year of ART and to evaluate antiretroviral pharmacokinetics and spatial drug distribution in relation to viral expression within LN tissue.

### Lymph node and blood sampling

For these objectives, 40 participants in the DUALITY study (20 per treatment arm) were invited to undergo a single inguinal LN excision during follow-up. Biopsy timepoints were randomly assigned within each arm at baseline (pre-ART) or at weeks 2, 4, 12 or 48 after ART initiation, with four participants per arm per timepoint. Venous blood sampling was performed in parallel to LN excision, approximately 24 hours after dosing. LNs were identified by ultrasound, and biopsies were conducted by experienced surgeons under local anesthesia using standard sterile techniques. A single LN was removed through a 1–2-cm skin incision, snap-frozen, and stored at −80°C together with plasma samples until analysis.

### Tissue sectioning

Each flash-frozen LN sample was adhered to a specimen disc with O.C.T. (Tissue-Tek; Sakura Finetek, Inc., Torrance, CA) and sectioned at −20°C using a Leica CM1950 cryomicrotome (Leica Biosystems, Buffalo Grove, IL). Subjacent LN sections (10μm) were thaw-mounted onto positively charged glass slides (IHC and ISH) or plain glass slides (IR-MALDESI). LN tissue from one participant (3DR, week 12) was damaged during cryosectioning and excluded from HIV DNA and RNA imaging but retained for drug distribution analyses.

### RNAscope/DNAscope ISH and multiplex IHC

ISH analysis of viral vRNA and vDNA was performed on subjacent LN sections using an adaptation of previously reported RNAscope protocols.^15,16^ Specifically, frozen tissues mounted on slides were thawed and dried at room temperature for 15 min and then dipped in phosphate-buffered saline (PBS) for 10 s prior to fixation. Slides were fixed by incubating in 4% paraformaldehyde (PFA) fixative (catalog number 15714-S; Electron Microscopy Sciences) in PBS for 2 h at room temperature followed by two 5 min incubations of 100% ethanol and then two rinses of double-distilled water (ddH_2_O). Heat-induced epitope retrieval was performed as follows: Slides were submerged in 1x RNAscope Target Retrieval Reagent (catalog number 322000; ACD) preheated to 97°C and then incubated and held at constant temperature for 5 min, and then immediately transferred to PBS. Slides were then incubated in peroxidase blocking solution (3% H_2_O_2_ diluted in 1x PBS) for 10 min at room temperature, followed by a ddH_2_O rinse.

Slides were incubated with clade specific target probes for HIV RNA or HIV DNA (Catalog numbers 416101, 416111, 429841, 446551, 426341, 425531, 430171, 478191; ACD) for 2h at 40°C. Amplification was performed using the RNAscope 2.5 red detection kit (catalog number 322360; ACD) using 0.5x wash buffer (catalog number 310091; ACD) for all washing steps. Slides were developed using Warp Red (catalog number WR806; Biocare) for 2 min.

Slides were then stained at room temperature using mouse anti-Collagen I (catalog number C2456; Sigma) (4.0 mg/mL) at 1:200 and goat anti-CD20 (catalog number PA1–9024; Invitrogen) (0.5mg/mL) at 1:200 overnight. The slides were then incubated with fluorescent secondaries donkey anti-mouse-AF647 (catalog number A31571; Invitrogen) (2 mg/mL) at 1:300 and donkey anti-goat-AF488 (catalog number A11055; Invitrogen) (2 mg/mL) at 1:200 for 1 h. Slides were counterstained with 4′,6-diamidino-2-phenylindole (DAPI) (catalog number D1306; Invitrogen) at a dilution of 0.5 μg/mL in ddH2O for 10 min and coverslipped using Prolong Gold antifade mounting medium (catalog number P36930; ThermoFisher), and whole tissues were imaged on an AxioScan.Z1 instrument (Zeiss) using a Plan Apochromat 20× objective (0.8 numerical aperture [NA], FWD = 0.55 mm; Zeiss). After fluorescent imaging, coverslips were removed and slides were counterstained with CAT hematoxylin (catalog number CATHE-GL; Biocare), mounted in Permount (Catalog number SP15–100; Fisher Scientific), and reimaged at 40x magnification on a brightfield Aperio AT2 (Leica Biosystems).

Manual annotations were drawn to define tissue regions and exclude areas of high artifact or morphological disruption from cryosectioning. HIV-infected cells were detected using the Object Colocalization v2.1.5 module within HALO image analysis software (v4.0; Indica Labs) using a minimum size threshold of 4.0 μm^2^ for RNA puncta and 0.5 μm^2^ for DNA puncta. Cell segmentation was performed using the Cytonuclear v1.6 module (v4.0; Indica Labs) to determine cellular density. Annotation pins were placed on putative HIV-infected cells based on anatomic localization and cell morphology. The fluorescent property of the Warp Red chromogen ensured that annotation pin identifiers matched cell-to-cell between brightfield and fluorescent images. Manual annotations based on CD20 staining were used to delineate the positions of B-cell follicles.

### LC-MS/MS and IR-MALDESI MSI analysis of antiretroviral drugs.

Concentrations of TFV, FTC, 3TC, DTG in plasma and in LN tissue homogenates along with the intracellular metabolites TFVdp, FTCtp, and 3TCtp were quantified using validated LC-MS/MS methods.^14,17,18^ TFV was monitored in plasma and tissue rather than TAF due to its longer half-life. Samples were extracted by protein precipitation with isotopically-labeled internal standards. Extracts were analyzed by a Shimadzu high-performance liquid chromatography system with analyte detection by a SCIEX API 5000 mass spectrometer. The lower limit of quantitation (LLOQ) in plasma was 1 ng/mL for all antiretrovirals. The LLOQs in tissue homogenates were 0.1ng/mL (TFV, FTC, 3TC, and DTG) and 0.2 ng/mL (TFVdp, FTCtp, 3TCtp). Sample concentrations were converted to units of ng/g or fmol/g for final reporting using the mass of tissue analyzed and the volume of the homogenization solvent. Assay precision and accuracy was within 15%. IF cell counts associated with each sample were used as estimates of tissue cell density for scaling intracellular metabolite concentrations to units of fmol/10^6^ cells for purposes of comparison to other studies.

Spatial distribution of antiretrovirals within LN was assessed by IR-MALDESI MSI.^9,11,23^ The technique utilizes an infrared laser to volatilize material from a focused region (a 100×100×10μm^3^ voxel), which is subsequently ionized by an electrospray before chemical analysis by a Thermo Fisher Scientific Q Exactive mass spectrometer (Bremen, Germany). Antiretroviral quantification by IR-MALDESI was determined by spotting calibration standards of known drug concentration onto LN tissue collected from individuals at week 0 prior to ART initiation. Intracellular metabolites could not be quantified by IR-MALDESI due to their degradation during sectioning.^14^ Per-voxel limits of detection were assessed based on the standard deviation of each analyte from replicate measurements of drug concentrations spotted onto blank tissue samples, and the slope of the calibration curve, with a signal-to-noise ratio of 3. Estimated IR-MALDESI LLOQs were 440 ng/g (TFV), 620 ng/g (FTC), 490 ng/g (3TC), and 240 ng/g (DTG).

### Image co-registration and colocalization analysis

Co-registration of MSI, ISH, and IHC images was performed in Matlab R2024a. Imported IF image data was color thresholded to isolate Collagen I staining, and then all image data and manual annotations of cell-associated vRNA and vDNA were down-sampled to match the spatial resolution of MSI data (pixel size: 100×100 μm) using the Image Processing Toolbox as described elsewhere.^9,11,23^ All image alignment was performed by manual selection of control points common to paired images of IF CD20 expression and an MSI endogenous lipid upregulated in B-cell follicles,^11^ followed by rigid registration (**Supplementary Fig. 4**). Colocalization analysis was constrained to tissue regions where ISH and IHC staining was successful. Spatial distributions of vRNA, vDNA, CD20, and collagen I were used to create binary masks associated with each marker, and additional masks of vRNA and vDNA were created that included all nearest-neighboring voxels to each location of vRNA + and vDNA+ cells. These masks were applied to MSI antiretroviral distributions to isolate the exposure of drug to each discrete target. To ensure that only drug exposure from parenchymal tissue was evaluated, spatial MSI antiretroviral response was corrected in regions there was colocalization with the blood marker heme.

### Statistical analysis.

Given that LN biopsy is an invasive procedure, each participant contributed a single LN biopsy; thus, data represent independent samples at each timepoint. Given these sampling constraints, we employed a naive pooled data analysis approach to describe changes in LN HIV-1 reservoir size over time. This approach allows comparison of distributions and trends across time points and treatment groups, under the assumption that biopsies at each time point are independent observations. Fold changes in HIV-1 DNA and RNA at week 48 relative to baseline were calculated as ratios of medians with 95% confidence intervals (95% CI) derived by nonparametric bias-corrected and accelerated (BCa) bootstrap resampling (1,000 iterations). For ratio calculations and graphical purposes, values below the LLOQ were set at 0.01 copies/10^5^ cells. Given the exploratory nature of this analysis and the limited sample size, no formal hypothesis testing between 2DR and 3DR arms was performed. Correlations between various HIV-1 reservoir measures and pharmacokinetic parameters in LN and peripheral blood were assessed using Spearman’s rank correlation with pooled data from all participants. The analysis was carried out using R project version 4.5.2. For graphical representations, GraphPad Prism version 11.0.0 was used.

## Supplementary Material

Supplementary Files

This is a list of supplementary files associated with this preprint. Click to download.

- DUALITYsupplementarymaterials.pdf

## Figures and Tables

**Table 1:** HIV-1 DNA- and RNA-positive cells in lymph nodes during the study.

	vDNA+ cells/10^5^ cells				vRNA+ cells/10^5^ cells			
	DTG+3TC		DTG + FTC/TAF		DTG+3TC		DTG + FTC/TAF	
	N	Median	N	Median	N	Median	N	Median
	(n BLQ)	(min-max)	(n BLQ)	(min-max)	(n BLQ)	(min-max)	(n BLQ)	(min-max)
Baseline (BL)	4 (0)	2.29 (0.80–4.14)	4 (0)	8.51 (0.51–36.86)	4 (0)	6.01 (1.37–13.03)	4 (0)	15.72 (0.62–38.41)
Week 2	4 (0)	0.43 (0.32–2.48)	4 (0)	2.39 (0.44–5.36)	4 (0)	1.07 (0.43–1.51)	4 (1)	0.61 (0.01–2.38)
Week 4	4 (0)	0.75 (0.26–1.48)	4 (0)	0.47 (0.15–1.27)	4 (1)	0.64 (BLQ – 0.95)	4 (2)	0.07 (BLQ – 0.19)
Week 12	4 (0)	0.29 (0.10–0.82)	3 (2)	BLQ (BLQ – 0.83)	4 (2)	0.05 (BLQ – 0.14)	3 (2)	BLQ (BLQ – 0.12)
Week 48	3 (0)	0.33 (0.14–0.59)	4 (0)	0.45 (0.20–1.83)	3 (1)	0.28 (BLQ – 1.46)	4 (3)	BLQ (BLQ – 1.10)
Ratio week48/BL*		0.158 (0.036–0.416)		0.049 (0.002–0.532)		0.055 (0.007–0.692)		0.001 (< 0.01–0.009)

* Expressed as median (95% CI).

Abbreviations: DTG, dolutegravir; 3TC, lamivudine; FTC, emtricitabine; TAF tenofovir alafenamide; BLQ, below lower limit of quantification.

**Table 2:** Antiretroviral drug concentrations in plasma and lymph nodes, measured by LC-MS/MS.

	N	Plasma	Lymph Node		LN/Plasma Ratio	LN:Plasma Correlation	LN Metabolite/Parent Ratio	LN Metabolite: Parent drug Correlation	Reference value
		ng/mL	μg/g	fmol/10^6^ cells		r, (p-value)		r, (p-value)	
**DTG**	29	1290 (620–3240)	0.261 (0.072–0.739)		0.18 (0.05–0.37)	0.819 (<0.0001)			64*
**3TC**	14	47.1 (31.1–88.9)	0.078 (0.001–0.286)		1.46 (0.02–6.46)	−0.08, (0.782)			
**3TCtp**			0.839 (0.008–1.886)	527 (5–1251)			4.19 (0.58–9.16)	0.688 (0.013)	564**
**FTC**	15	58.7 (40.0–150.0)	0.114 (0.012–0.368)		1.58 (0.24–5.20)	0.278 (0.336)			
**FTCtp**			0.342 (0.026–1.652)	258 (15–990)			1.59 (0.57–5.73)	0.366 (0.198)	338**
**TFV**	15	9.8 (4.4–14.2)	0.044 (0.022–0.108)		4.34 (2.06–11.62)	0.277 (0.340)			
**TFVdp**			0.145 (0.049–0.550)	104 (35–395)			1.58 (0.57–4.94)	0.516 (0.059)	51***

Data are expressed as median (min-max);

* IC90 (ng/mL);

** IC50 (fmol/10^6^ cells);

*** EC50 (fmol/10^6^ cells)

Abbreviations: LN, lymph node; DTG, dolutegravir; 3TC, lamivudine; 3TCtp, lamivudine triphosphate; FTC, emtricitabine; FTCtp, emtricitabine triphosphate; TFV, tenofovir; TFVdp, tenofovir diphosphate.

## Data Availability

The study protocol is available in the Supplementary Information file. Raw clinical data are not publicly available due to data privacy regulations. De-identified individual and/or study-level data are available from the corresponding authors upon reasonable request and if regulatory criteria are met.
